# Significance of HLA-E and its two NKG2 receptors in development of complications after allogeneic transplantation of hematopoietic stem cells

**DOI:** 10.3389/fimmu.2023.1227897

**Published:** 2023-10-13

**Authors:** Jagoda Siemaszko, Piotr Łacina, Donata Szymczak, Agnieszka Szeremet, Maciej Majcherek, Anna Czyż, Małgorzata Sobczyk-Kruszelnicka, Wojciech Fidyk, Iwona Solarska, Barbara Nasiłowska-Adamska, Patrycja Skowrońska, Maria Bieniaszewska, Agnieszka Tomaszewska, Grzegorz W. Basak, Sebastian Giebel, Tomasz Wróbel, Katarzyna Bogunia-Kubik

**Affiliations:** ^1^ Laboratory of Clinical Immunogenetics and Pharmacogenetics, Hirszfeld Institute of Immunology and Experimental Therapy, Polish Academy of Sciences, Wroclaw, Poland; ^2^ Department of Hematology, Blood Neoplasms and Bone Marrow Transplantation, Wroclaw Medical University, Wroclaw, Poland; ^3^ Department of Bone Marrow Transplantation and Hematology-Oncology, Maria Sklodowska-Curie Memorial Cancer Center and Institute of Oncology, Gliwice, Poland; ^4^ Institute of Hematology and Blood Transfusion Medicine, Warsaw, Poland; ^5^ Cell and Tissue Bank, University Medical Center in Gdansk, Gdansk, Poland; ^6^ Department of Hematology and Transplantology, Medical University of Gdansk, Gdansk, Poland; ^7^ Department of Hematology, Transplantation and Internal Medicine, Medical University of Warsaw, Warsaw, Poland

**Keywords:** HSCT, NK cells, NK cell receptors, NKG2A, NKG2C, HLA-E, sHLA-E, transplant outcome

## Abstract

Transplantation of hematopoietic stem cells (HSCT) is a procedure commonly used in treatment of various haematological disorders which is associated with significantly improved survival rates. However, one of its drawbacks is the possibility of development of post-transplant complications, including acute and chronic graft-versus-host disease (GvHD) or CMV infection. Various studies suggested that NK cells and their receptors may affect the transplant outcome. In the present study, patients and donors were found to significantly differ in the distribution of the *NKG2A* rs7301582 genetic variants – recipients carried the *C* allele more often than their donors (0.975 vs 0.865, p<0.0001). Increased soluble HLA-E (sHLA-E) levels detected in recipients’ serum 30 days after transplantation seemed to play a prognostic and protective role. It was observed that recipients with higher sHLA-E levels were less prone to chronic GvHD (11.65 vs 6.33 pg/mL, p=0.033) or more severe acute GvHD grades II-IV (11.07 vs 8.04 pg/mL, p=0.081). Our results also showed an unfavourable role of HLA-E donor-recipient genetic incompatibility in CMV infection development after transplantation (OR=5.92, p=0.014). Frequencies of NK cells (both CD56dim and CD56bright) expressing NKG2C were elevated in recipients who developed CMV, especially 30 and 90 days post-transplantation (p<0.03). Percentages of NKG2C+ NK cells lacking NKG2A expression were also increased in these patients. Moreover, recipients carrying a *NKG2C* deletion characterized with decreased frequency of NKG2C+ NK cells (p<0.05). Our study confirms the importance of NK cells in the development of post-transplant complications and highlights the effect of HLA-E and NKG2C genetic variants, sHLA-E serum concentration, as well as NKG2C surface expression on transplant outcome.

## Introduction

Allogeneic haematopoietic stem cell transplantation (HSCT) is a standard form of treatment for patients diagnosed with haematological disorders, including malignancies. Even though it is a common procedure, it may lead to development of serious complications, such as graft-versus-host disease (GvHD), which may occur in an acute (aGvHD) or chronic (cGvHD) form. Acute GvHD is initiated as a reaction of donor cells towards tissues of the recipient. It affects skin, gut, lung and liver depending on the severity of the disease, which is graded I to IV. Chronic GvHD has a different pathogenesis and can affect more organs than aGvHD (1, 2). Aside from GvHD, another type of post-transplant complications is viral infections, particularly those caused by Herpesviridae such as cytomegalovirus (CMV) or Epstein-Barr virus (EBV). Asymptomatic, latent CMV infections are extremely common, with as many as 50% of people being seropositive for CMV (3). However, CMV reactivation may be life-threatening in immunocompromised persons, such as post-HSCT patients (4).

Natural killer (NK) cells, as the first donor-delivered lymphocytes to reconstitute after HSCT, have protective properties against GvHD with a simultaneous ability to induce a graft-versus-leukaemia (GvL) effect (5–7). NK cells are orchestrated by a wide set of activating and inhibitory receptors, whose ligands are classical and non-classical Major Histocompatibility Complex (MHC) molecules, e.g. HLA-E. In contrast to other MHC molecules, HLA-E is very conserved and its polymorphism is mostly limited to two major alleles, *01:01 and *01:03, comprising over 99% of allele frequency globally (8). Both of these alleles are distributed with similar frequencies and differ in a single Arg/Gly substitution in position 107. Other *HLA-E* alleles exist, although they are extremely rare. Some of these alleles also characterize with alternative substitutions in position 107 (e.g. HLA-E*01:48, with allele frequency of 0.0007%) (9). HLA-E serves as a ligand for two NKG2 receptors, inhibitory NKG2A and activating NKG2C (encoded by the *KLRC1* and *KLR2C* genes, respectively). It can also be secreted in a soluble form (sHLA-E). This soluble form may play a role in immune regulation (10), and sHLA-E levels are increased in various cancers and autoimmune diseases (11–14). HLA-E:NKG2A/C interactions are essential for balancing the NK cell reactivity (15). Despite the molecular and structural similarities of these two receptors, HLA-E binds NKG2A with 6-fold higher affinity, what helps to monitor the expression of the MHC class I molecules on normal cells (16). NKG2C expression is low in immature NK cells, and then subsequently increases during maturation, while NKG2A expression concurrently decreases (17). A specific subset of NKG2C+ cells has been observed to expand in response to cytomegalovirus (CMV) reactivation, but not other viral infections such as EBV. These cells can function like adaptive memory cells, and, if transplanted from CMV seropositive donors, exhibit a heightened response to a secondary CMV event (18–21). They persist for a long time after infection and lack NKG2A expression (20). NKG2C+ NK cells were also observed to interact with CMV-specific CD8+ T cells to combat CMV infection (22). Both NKG2A and NKG2C are minimally polymorphic compared to classical HLA genes. There are several rarely studied single nucleotide polymorphisms (SNPs) in the *NKG2C* gene, some of which are located in coding regions, associated with three alleles designated as NKG2C*01, NKG2C*02 and NKG2C*03 (23, 24). In contrast, a major NKG2C deletion resulting in a loss of expression or reduced expression (in homo- and heterozygotes, respectively) has been far better studied and is well known for its importance in viral infections (25). As reported in many studies (26–28), patients carrying at least one *del* variant are more susceptible for CMV (especially reactivation after HSCT), and HIV infections, nonetheless the studies are not always consisted (29). It has been recently suggested that the *NKG2C* deletion may also increase risk for SARS-CoV-2 infections, although this seems to require further validation (30).

The selected *HLA-E* rs1264457 SNP is localized in the third exon. It results in a *T* (*01:01)/*C* (*01:03) nucleotide substitution associated with Gly to Arg amino acid exchange. The selected polymorphism for the NKG2A inhibiting receptor was rs7301582, an intronic *C*/*T* substitution. Both SNPs (*HLA-E* rs1264457 and *NKG2A* rs7301582) have also been studied in our recent study on post-transplant complications in paediatric HSCT recipients (31), while in our previous work on patients with inflammatory (rheumatoid) arthritis, they were described to be associated with response to anti-TNF treatment (32, 33). Another authors investigated these two SNPs in their studies, as e.g. Kordelas et al., who proved a protective effect of HLA-E*01:03 homozygosity in overall survival after HSCT (34, 35).

We hypothesize that HLA-E, NKG2A and NKG2C expression and polymorphism play a role in the development of complications after HSCT in a Polish population. Various studies previously described the role of HLA-E and NKG2A/C polymorphisms in different population groups, although their results were not always consistent (28, 36–44). In our present study, we aimed to determine the role of *HLA-E* genetic polymorphism and soluble HLA-E concentration, as well as *NKG2A* and *NKG2C* gene polymorphisms and protein expression within the NK cells in the development of post-transplant complications in recipients of allogeneic hematopoietic stem cells.

## Materials and methods

The study group consisted of 200 HSCT recipients (aged 19-73) and 104 of their donors treated in five Polish transplantation centres. Patients were assigned for allogeneic HSCT according to European Society for Blood and Marrow Transplantation criteria. Exclusion criteria were: age < 18 years old, high Haematopoietic Cell Transplantation-specific Comorbidity Index (HCT CI), and Karnofsky index < 80%. The most common haematological disease was acute myeloid leukaemia (AML), diagnosed in 42.5%, followed by acute lymphoblastic leukaemia (ALL), diagnosed in 13.5% of recipients. The patients characteristics and transplant details are presented in **Table 1**. Recipients and donors were genotyped at high resolution level for at least 5 HLA loci (HLA-A, B, C, DRB1 and DQB1). This study complies with the Declaration of Helsinki and was approved by the Wroclaw Medical University Ethics Committee (identification code KB-561/2019).

**Table 1:** Patients’ characteristics and transplant details.

Clinical data	Number of patients
**N**	200
**Age median (range)**	52 (19-73)
Recipient sex	
**M/F**	117/83 (58.50%/41.50%)
Diagnosis	
**AML**	85 (42.5%)
**ALL**	27 (13.5%)
**MDS**	24 (12%)
**MPN**	18 (9%)
**NHL**	17 (8.5%)
**HL**	9 (4.5%)
**PCM**	6 (3%)
**Other**	14 (7%)
Type of donor	
**MUD** **MMUD**	45 (22.5%) 13 (6.5%)
**MSD**	96 (48%)
**haploidentical**	56 (28%)
Donor/Recipient sex match	
**M/M**	88 (44%)
**M/F**	47 (23.5%)
**F/M**	28 (14%)
**F/F**	33 (16.5%)
Donor/Recipient CMV status match	
**+/+**	130 (65%)
**+/-**	16 (8%)
**-/+**	35 (17.5.%)
**-/-**	19 (9.5%)
Conditioning	
**MAC**	107 (53.5%)
**RIC**	86 (43%)
**NMA**	3 (1.5%)
Post-transplant complications	
**aGvHD grades I-IV**	81 (40.5%)
**aGvHD grades II-IV**	42 (21%)
**cGvHD**	42 (21%)
**CMV infection**	81 (40.5%)
**Relapse**	23 (11.5%)
**Death**	23 (11.5%)

AML, acute myeloid leukaemia; ALL, acute lymphoblastic leukaemia; MDS, Myelodysplastic Syndrome; MPN, myeloproliferative neoplasm; PCM, plasma cell myeloma; HNL, non-Hodgkin lymphoma; HL, non-Hodgkin lymphoma; Other (including Paroxysmal Nocturnal Haemoglobinuria; Blastic Plasmacytoid Dendritic Cell Neoplasm); MUD, matched unrelated donor; MMUD, mismatched unrelated donor; MSD, matched sibling donor; MAC, myeloablative conditioning; RIC, reduced intensity conditioning; NMA, non-myeloablative conditioning; CMV, cytomegalovirus.

### Soluble HLA-E measurement

Serum from 102 recipients was used for measurement of sHLA-E concentration. Serum sHLA-E was measured with use of the commercially available enzyme-linked immunosorbent assay (ELISA) kit (ELK Biotechnology, USA, Cat.No. ELK2168). Experiment was performed following the manufacturer’s protocol. Absorbance was measured at λ=450nm using Sunrise microplate reader (Tecan, Switzerland).

### SNP genotyping

For the genetic studies, whole blood of the HSCT recipients and their donors was collected into ethylenediaminetetraacetic acid (EDTA) tubes. Genomic DNA extraction was performed using NucleoSpin Blood kit (MACHEREY-NAGEL, Germany, Cat.No. 740951.50) following the manufacturer’s protocol. Two single nucleotide polymorphisms were chosen based on literature analysis and the online SNPinfo Web Server prediction tool (45). SNPs detection was performed with the use of LightSNiP assays (TibMOLBIOL, Switzerland) and carried out in a LightCycler 480 II instrument (Roche Applied Science, Germany) with melting curves analyses. A negative control using PCR-grade water instead of DNA was included in all experiments.

### *NKG2C* deletion

The *NKG2C/KLRC2 wt*/*del* variants were determined using PCR-SSP with two pairs of oligonucleotides as previously described (46, 47). First pair of primers (KLRdelF 5’-ACTCGGATTTCTATTTGATGC-3’ and KLRdelR 5’-ACAAGTGATGTATAAGAAAAAG-3’) is specific for *NKG2C* deletion while second pair (KLRFg669 5’-CAGTGTGGATCTTCAATG-3’ and KLRR+135 5’-TTTAGTAATTGTGTGCATCCTA-3’) amplifies in the presence of *NKG2C* gene. The PCR was performed with the use of Multiplex Master Mix (EURx, Poland, Cat. No. E2820-01) at following conditions: 10 min of initial denaturation at 95°C, then 40 cycles of 30s denaturation at 94°C, 90s annealing at 56°C and 30s extension at 72°C, then 7min of final extension at 68°C. For *NKG2C wt*/*del* detection 10 µl of final PCR products were electrophoresed in 2% agarose gels with 1x TBE buffer stained with SimplySafe™ (EURx, Poland, Cat.No. E0301-100, E0230-01 and E4600-01) and then visualized by the UV exposure.

### Flow cytometry analysis

For the flow cytometer analysis, 10 mL of peripheral blood, collected in EDTA tubes (BD), was used. Recipients’ blood was collected before HSCT and at four time points after transplantation; +21, +30, +60 and +90 days. Cells were surface stained in one eight-colour tube. The following mouse anti-human monoclonal antibodies, all purchased from Becton Dickinson and Company (BD), San Jose, CA, were used for analysis: CD94 (BD Pharmingen™ FITC, Clone HP-3D9, Cat.No. 555888), NKG2C (CD159c, BD OptiBuild™ BB700, Clone 134591, Cat.No. 748162), CD56 (BD Pharmingen™ APC, Clone B159, Cat.No. 555518), CD3 (BD Pharmingen™ APC-H7, Clone SK7, Cat.No. 560176), NKG2A (CD159a, BD OptiBuild™ BV421, Clone 131411, Cat.No. 747924), CD16 (BD Horizon™ V500, Clone 3G8, Cat.No. 561393 Lysing solution BD was used for lysing (diluted 10 times). The evaluation of nucleated cells was carried out on an 8-color FACS Canto II flow cytometer (BD).

**Figure 1:**
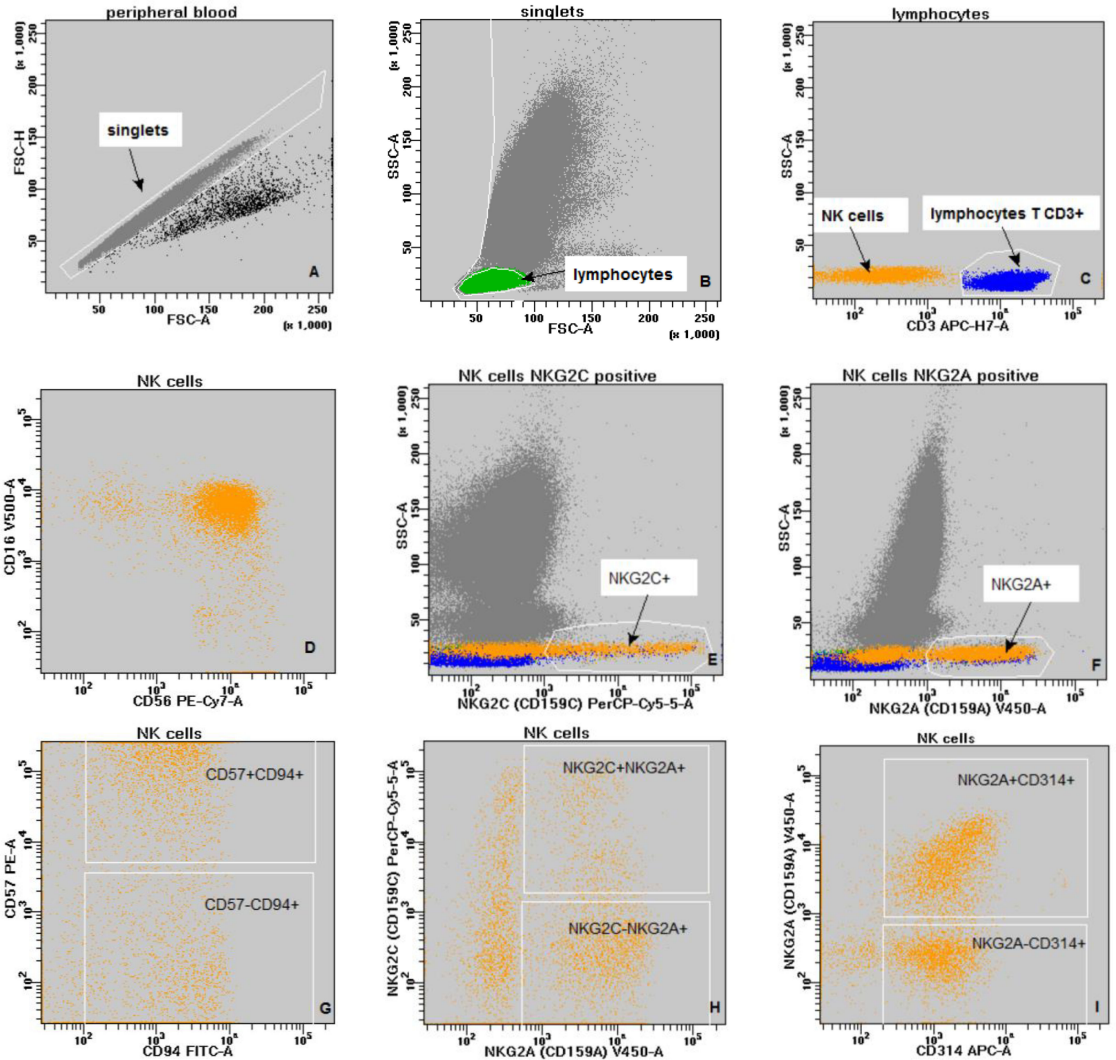
The gating strategy **(A–G)**: grey - singlets; green - lymphocytes; blue - lymphocytes T CD3+; orange - NK cells. **(A)** - discrimination of doublets (FSC-A vs. FSC-H); **(B)** - discrimination of debris and lymphocytes gating; (FSC-A vs. SSc-A); **(C)** - lymphocyte subpopulations: lymphocytes T CD3+ (blue) and NK cells (orange) - (CD3 vs. SSc-A); **(D)** - NK cells (CD56 vs. CD16); **(E)** - NK cells NKG2C positive (NKG2C vs. SSc-A); **(F)** - NK cells NKG2A positive (NKG2A vs. SSc-A); **(G)** - NK cells double positive CD94+CD57+ (CD94 vs. CD57); **(H)** - NK cells double positive NKG2A+NKG2C+ (NKG2A vs. NKG2C); **(I)** - NK cells double positive CD314+NKG2A+ (CD314 vs. NKG2A).

The gating strategy for the assessment of NKG2 receptor on NK cells is shown below (**Figure 1**).

### Statistical analysis

Genotype and allele frequencies of studied SNP’s were calculated using the Fisher’s exact test. For analyses related to sHLA-E concentration, the nonparametric Mann–Whitney test for continuous variables was used. Logistic regression model was used for multivariate analysis of CMV infection risk. Programs used for data visualisations were RStudio v.4.2 and GraphPad Prism v.5.0. The flow cytometer data were analysed with the use of BD FACSDiva software v8.0.ric. A p-value < 0.05 was considered statistically significant. All alleles were in the Hardy Weinberg equilibrium, both in recipients and in donors.

## Results

### Donor-recipient genotyping – differences in *NKG2A* genotype distribution between patients and donors

**Figure 2:**
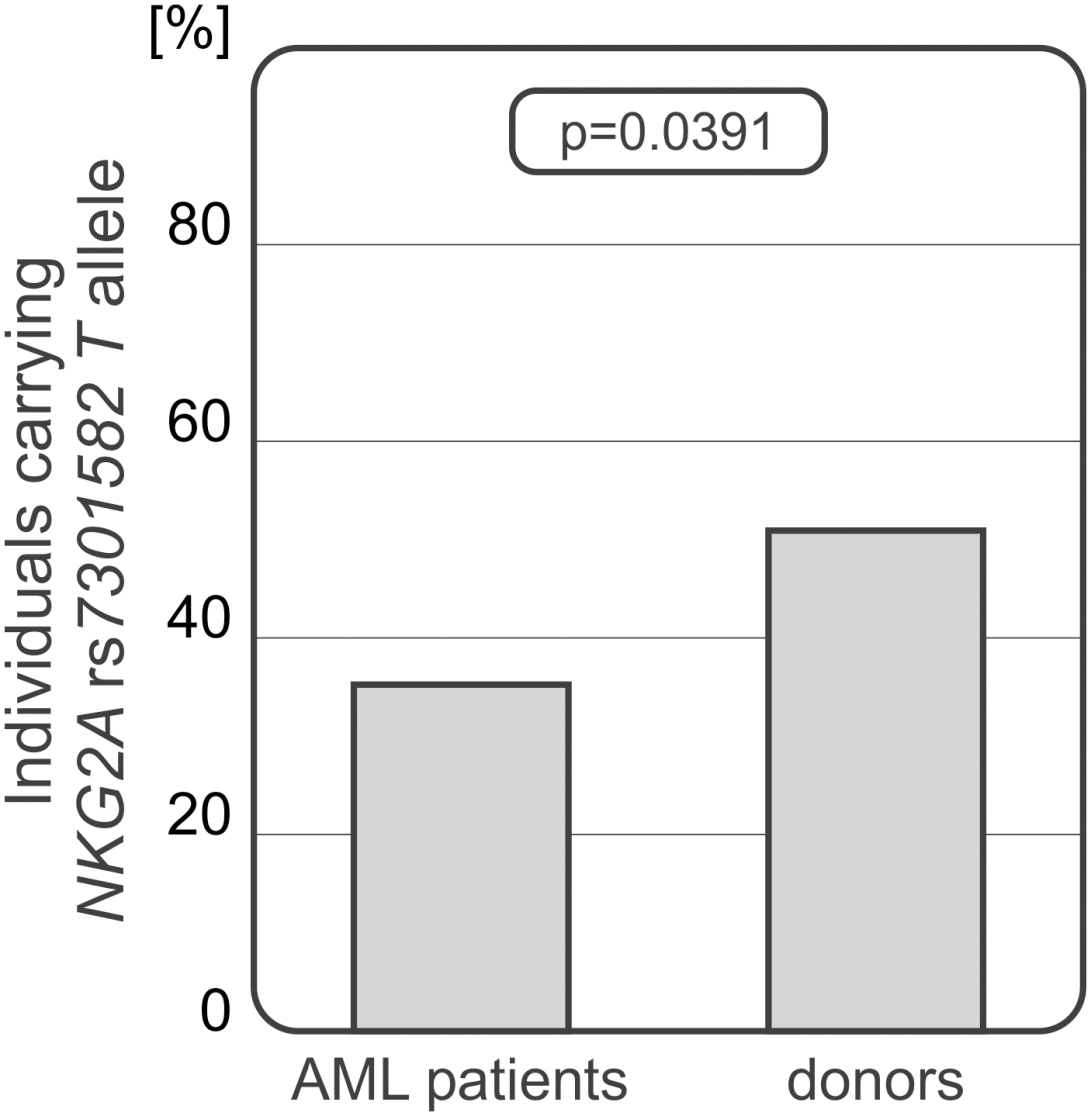
Differences in *NKG2A* rs7301582 *T* allele frequencies between AML patients and donors.

Frequency of recipient and donor genotypes and alleles are shown in **Table 2**. Donors and HSCT recipients did not differ in genotype or allele distribution of *HLA-E* rs1264457 polymorphism and *NKG2C wt*/*del* variants. A significant difference was seen in genotype distribution of *NKG2A* rs7301582 polymorphism between recipients and donors. Recipients carried the *CC* genotype (65.5% vs 49.04%, OR=1.97; 95%CI 1.218 – 3.197, p<0.0001) and *C* allele (97.50% vs 86.54%, OR=2.09, 95%CI 1.424-3.077, p<0.0001) more frequently than their donors. The latter relationship was also seen when the patients with AML and group of healthy donors were considered. The patients diagnosed with AML (85 recipients, 42.5% of all patients) characterized with decreased frequency of *NKG2A* rs7301582 *T* allele when compared to donor group (35.29% vs 50.96%, p=0.039) (**Figure 2**).

**Table 2:** Distribution of genetic variants in patients and HSCT donors.

		Recipients (N=200)	Donors (N=104)	P value
NKG2A rs7301582				
***genotypes***	***CC***	131(65.50%)	51 (49.04%)	< 0.0001^1^
	***CT***	64 (32.00%)	39 (37.50%)	
	***TT***	5 (2.50%)	14 (13.46%)	
***alleles***	***C***	326 (81.50%)	141 (67.79%)	< 0.0001
	***T***	74 (18.50%)	67 (32.21%)	
HLA-E rs1264457				
***genotypes***	***CC***	37 (18.5%)	21 (20.19%)	0.759^2^ 0.900^3^
	***CT***	92 (46%)	47 (45.19%)	
	***TT***	71 (35.5%)	36 (34.62%)	
***alleles***	***C***	166 (41.5%)	89 (42.79%)	0.795
	***T***	234 (58.5%)	119 (57.21%)	
*NKG2C* deletion				
***genotypes***	***wt/wt***	134 (67%)	72 (69.23%)	0.701^4^ 1^5^
	***wt/del***	63 (31.5%)	29 (27.88%)	
	***del/del***	3 (1.5%)	3 (2.88%)	
***alleles***	***wt***	331 (82.75%)	173 (83.17%)	0.910
	***del***	69 (17.25%)	35 (16.83%)	

^1^ *CC/CT* vs *TT* and *CC* vs *CT/TT*, ^2^ *CC* vs *CT/TT*, ^3^ *CC/CT* vs *TT*, ^4^ *wt/wt+wt/del* vs *del/del*, ^5^ *wt/wt* vs *wt/del + del/del*.

### Donor-recipient matching – role of HLA-E mismatch

**Figure 3:**
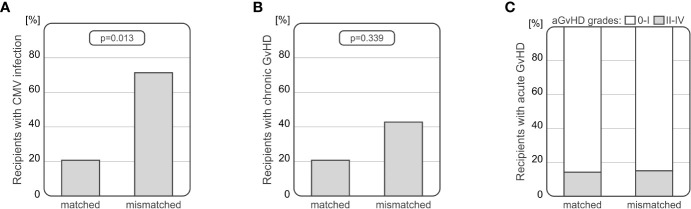
Associations between HLA-E incompatibility and post-transplant complications development; CMV infection **(A)** and chronic GvHD **(B)** occurred more often among patients after HLA-E mismatched HSCT. Acute GvHD was developed irrespective of HLA-E compatibility **(C)**.

Interestingly, donor-recipient HLA-E matching seems to have an impact on HSCT outcome. Among 60 10/10 HLA matched donor/recipient pairs, 7 pairs were mismatched in regards to the *HLA-E* rs1264457 SNP. We observed that the CMV infection occurred more frequently among recipients transplanted with HLA-E mismatched donors (5/7 cases, 71.43%) than after HLA-E matched transplantation (11/53, 20.75%, p=0.013, **Figure 3A**). A model using multivariate logistic regression including *NKG2C* genotype, HLA mismatch, donor and recipient CMV status, donor and recipient sex as well as age showed that HLA-E mismatch is an independent marker of CMV infection (p=0.014, OR=5.92, 95%CI 1.57 - 29.22). Furthermore, this analysis also confirmed the prognostic value of recipient CMV status (p=0.003, OR=35.90, 95%CI 4.89 - 812.49).

No associations were observed in analyses performed separately for AML and ALL patients (p>0.05). Interestingly, symptoms of chronic GvHD were observed among recipients with HLA-E mismatched donors more frequently (3/7 cases, 42.86%) than among matched pairs (11/53 cases, 20.75%), however, this difference did not rich statistical significance (p=0.339, **Figure 3B**). No associations between HLA-E compatibility and the risk of acute GvHD were observed (**Figure 3C**).

### Serum sHLA-E concentration

**Figure 4:**
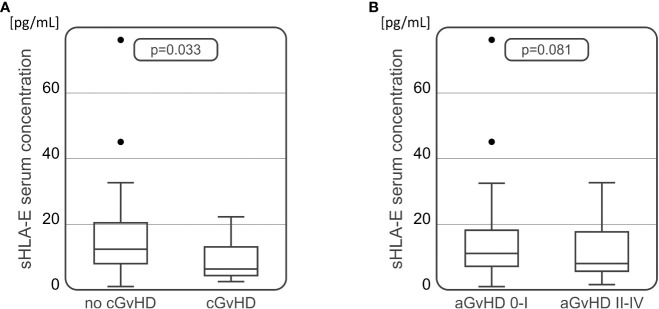
Soluble HLA-E levels detected in recipients’ serum 30 days after transplantation were lower in recipients who developed chronic **(A)** or acute **(B)** GvHD in comparison to recipients without these complications.

For the soluble HLA-E level measurements, 102 samples of serum collected 30 days after HSCT were used. Median serum sHLA-E concentration in the recipient group was 9.92 pg/mL (**Table 3**). The HSCT recipients diagnosed with cGvHD characterized with significantly decreased sHLA-E level in comparison to those without cGvHD symptoms (6.33 vs 11.65 pg/mL, p=0.033, **Figure 4A**). In recipients suffering from aGvHD grades II-IV, median sHLA-E level was also decreased when compared to the recipients without aGvHD symptoms or diagnosed with mild grade I, but this difference was not statistically significant (8.04 vs 11.07 pg/mL, p=0.081, **Figure 4B**).

**Table 3:** Median (with IQR) serum sHLA-E concentration values in different post-transplant conditions.

	n	+30 days [pg/mL]	*P* value	n	+90 days [pg/mL]	*P* value
**All recipients**	102	9.92 (6.30-18.68)		40	29.20 (24.22-40.04)	
**No cGvHD**	84 (82.35%)	11.65 (6.75-20.31)	0.033	20 (50%)	29.31 (17.82-36.26)	0.376
**cGvHD**	18 (17.65%)	6.33 (4.09-14.74)		20 (50%)	29.10 (24.28-44.87)	
**aGvHD 0-I**	81 (79.41%)	11.07 (6.5-19.32)	0.081	33 (82.5%)	28.94 (22.33-39.78)	0.512
**aGvHD II-IV**	21 (20.59%)	8.04 (5.44-18.19)		7 (17.5%)	29.27 (24.63-42.14)	
**CMV**	37 (36.27%)	9.01 (6.31-20.73)	0.645	26 (65%)	29.27 (19.79-43.86)	0.512
**No CMV**	65 (63.73%)	9.93 (5.67-16.14)		14 (35%)	28.94 (24.14-37.27)	
**Age <52**	48 (47.06%)	10.55 (6.29-19.63)	0.134	20 (50%)	29.20 (21.91-39.25)	0.883
**Age >52**	54 (52.94%)	8.79 (5.65-18.1)		20 (50%)	29.01 (23.99-39.59)	

**Figure 5:**
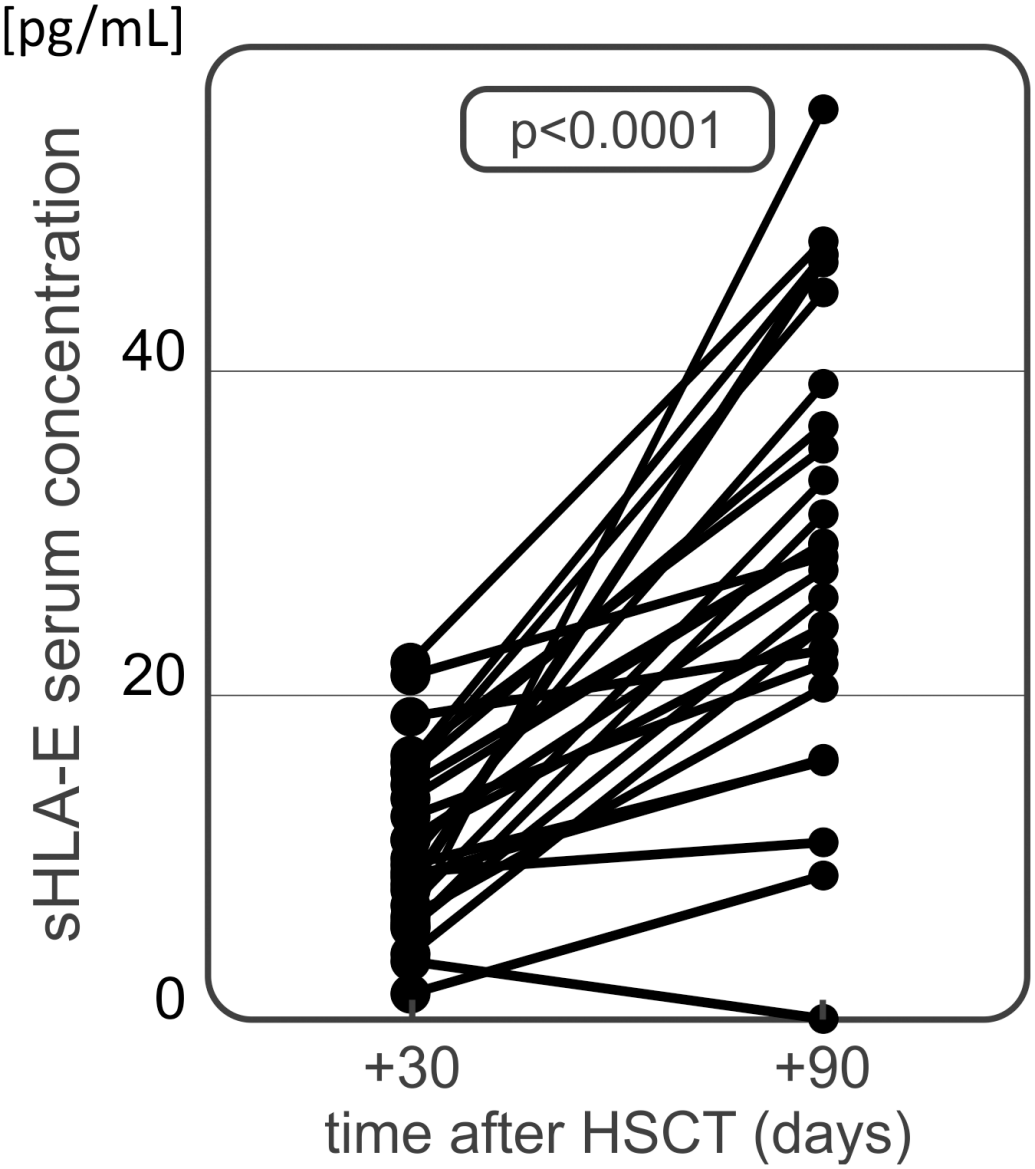
Serum sHLA-E concentration in HSCT recipients at day 30 and 90 after transplantation. A significant time-dependent increase has been observed.

An additional analysis of sHLA-E concentration was performed on 40 serum samples collected from patients 90 days after transplantation. The median serum sHLA-E concentration equalled 29.20 pg/mL 90 days after transplantation. No differences were found in sHLA-E levels measured in this time point between patients having and lacking various post-transplant complications (**Table 3**). This included 30 patients from whom the samples were also collected 30 days after HSCT. In this group of patients we observed a significant increase in sHLA-E level at day 90 after transplantation as compared to day 30 (9.93 vs 29.31 pg/mL, p<0.001, **Figure 5**). Similar difference was seen when we compared all the samples collected at 30 (n=102) and 90 (n=40) day after HSCT (9.92 vs 29.20 pg/mL, p<0.001). No associations were seen when sHLA-E serum concentration was compared to the results of *HLA-E* rs1264457 genotyping.

### *NKG2C* deletion and sHLA-E serum level

**Figure 6:**
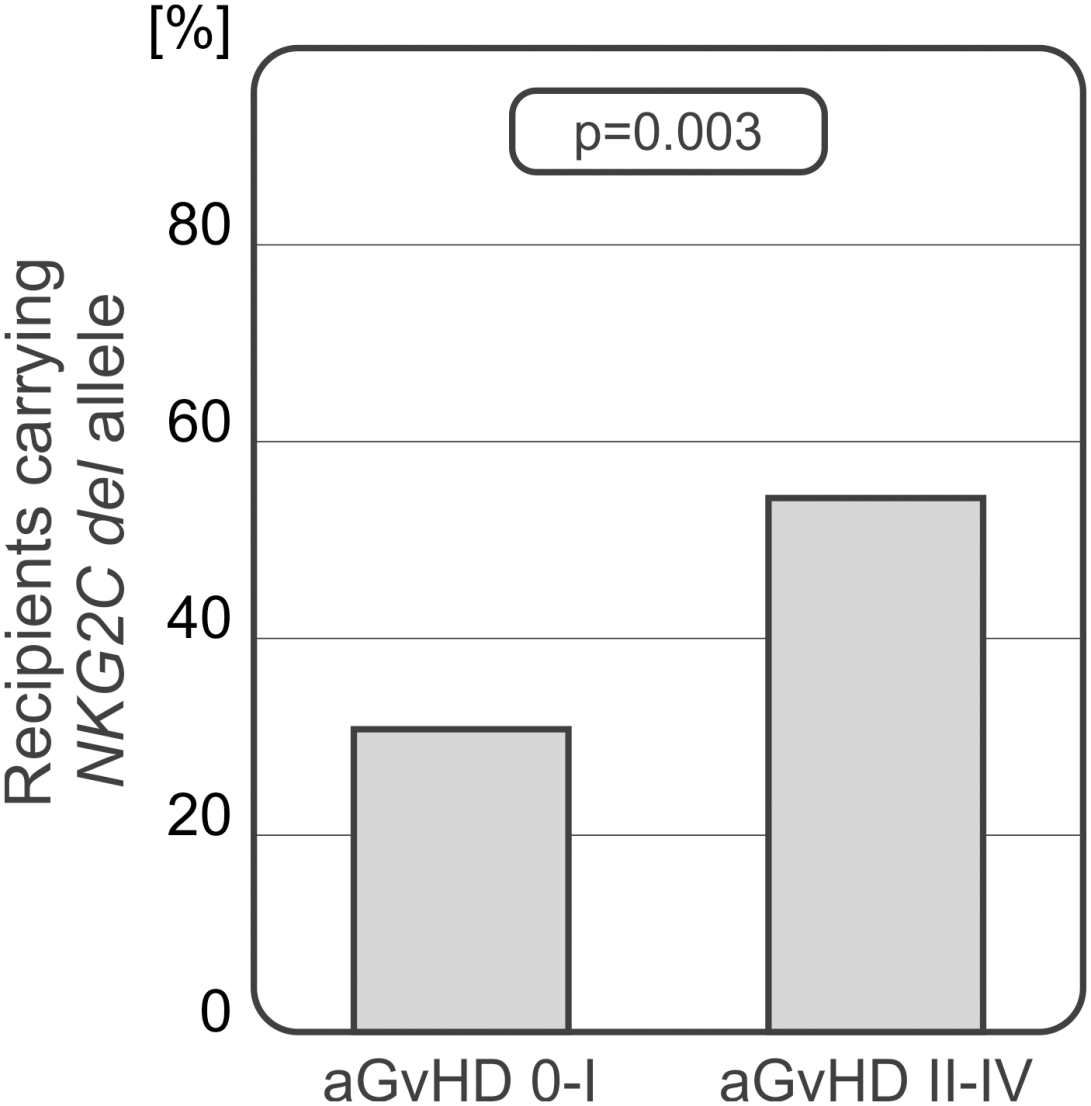
Recipients who developed severe acute GvHD grades II-IV carried the *NKG2C del* variant more frequently.

The *NKG2C* deletion analysis showed that patients diagnosed with more severe aGvHD grades II-IV carried the *del* variant more frequently. Recipients who did not develop aGvHD or were diagnosed with grade I of the disease, characterised with decreased frequency of the *del* variant which was detected more often in patients with severe II-IV aGvHD (25.93% vs 51.61%, p=0.003, **Figure 6**).

Interestingly, a potential relationship between the *NKG2C* genotype and sHLA-E serum concentration was observed. Recipients carrying at least one *NKG2C del* allele (*wt*/*del* or *del*/*del* genotypes) seemed to characterize with increased sHLA-E levels. Their median serum sHLA-E concentration was 15.248 pg/mL, while in *wt*/*wt* homozygotes this value equalled 9.923 pg/mL (p=0.332).

### Expression of NKG2C on NK cells

Peripheral blood of 28 HSCT recipients was used for flow cytometry analysis of NK cells expressing NKG2A and NKG2C. The analysis focused on all NK cells as well as their subsets (NK CD56dim and NK CD56bright cells), based on expression of CD3, CD16 and CD56 markers. Differences in expression of NKG2 receptors were assessed and compared at various time points before (day 0) and after (days +21, +30, +60 and +90) transplantation and related with the transplant outcome. Some statistically significant associations were detected with respect to the development of CMV infection and the frequency of NK cells expressing NKG2C. The average onset of CMV infection in this cohort was 44 days after HSCT.

**Figure 7:**
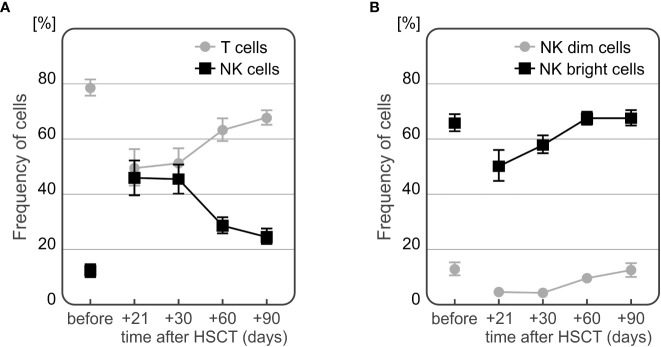
Changes in frequencies of T and NK cells **(A)** and NK cell subpopulations **(B)** during the observation period.

Recipients presented with a higher frequency of NK cells directly after HSCT, which decreases over time with a simultaneous increase of T cell percentage. As expected the frequency of NK CD56bright cells was higher than that observed of the NK CD65dim subpopulation (**Figure 7**).

**Figure 8:**
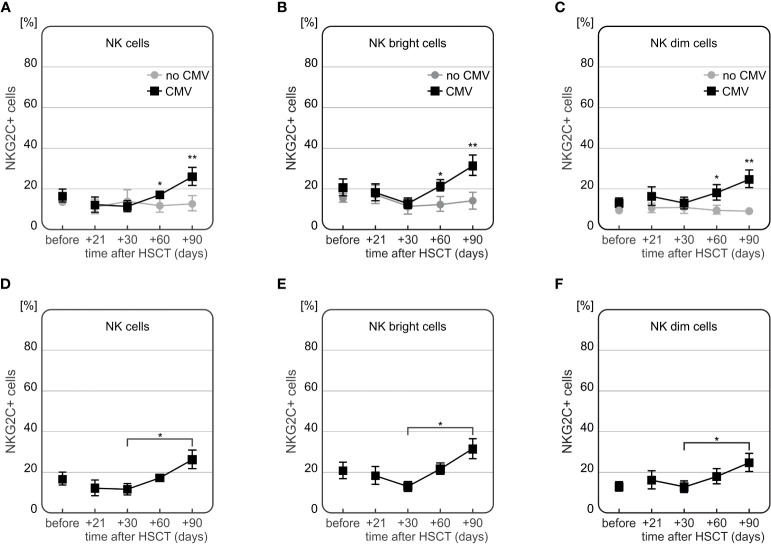
Differences in expression of NKG2C on NK cells in HSCT recipients with CMV infection at various time points after transplantation. Frequency of NKG2C+ cells was increased 60 (*p=0.005) and 90 (**p=0.007) days after HSCT in patients who developed CMV infection as compared to patients without this complication **(A)**. Frequency of NKG2C+ NK CD56bright cells was higher among recipients with CMV infection (day +60 *p=0.006 and day +90 **p=0.009, **(B)**. Similarly, frequency of NKG2C+ NK CD56dim cells was increased in recipients with CMV infection (day +60 *p=0.025 and day +90 **p=0.003, **(C)**. Patients with CMV infection characterized with a significant increase in frequency of NK cells expressing NKG2C cells between days 30 and 90 after HSCT (*p=0.016, **D**). A significant difference between days 30 and 90 after HSCT was also observed for the frequencies of NKG2C+ NK CD56bright cells (*p=0.008, **E**) as well as NKG2C+ NK 56dim cells (*p=0.030, in patients with CMV infection **F**).

The frequency of NKG2C+ NK cells was significantly higher in recipients who developed CMV infection after HSCT (**Figures 8A–C**). At days +60 and +90 day after HSCT, recipients with CMV infection (n=13) characterized with increased frequency of NKG2C+ cells compared to individuals without infection (n=15). This association was observed in both CD56bright and CD56dim NK cell subsets (p=0.006 and p=0.009 for the frequency of NK CD56bright cells expressing NKG2C at day +60 and day +90, respectively, and p=0.025 and p=0.003 for the frequency of NK CD56dim cells expressing NKG2C at day +60 and day +90, respectively).

**Figure 9:**
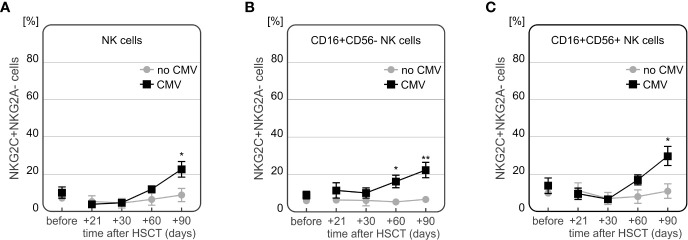
Differences in expression of NKG2A-/NKG2C+ NK cells in patients with CMV infection at various time points after transplantation. Increased frequency of NKG2A-/NKG2C+ NK cells in recipients with CMV infection 90 days after HSCT (p=0.003, **A**). Frequency of NKG2A-/NKG2C+ CD56bright NK cells was significantly increased in CMV patients 30 and 90 days after transplantation (p=0.002 and p=0.002, respectively, **B**). Higher frequency of NKG2A-/NKG2C+ population of CD56dim NK cells was observed in 90 days after HSCT (p=0.004, **C**).

Moreover, a time-dependent increase in frequency of NKG2C+ NK cells after HSCT was observed in CMV patients (n=7). These recipients characterized with the highest frequency of NKG2C+ NK cells 90 days after transplantation, especially as compared to day +30 post HSCT (**Figures 8D–F**). An additional analysis of the NKG2A-/NKG2C+ NK cell subpopulation showed that it was more frequently detected in recipients who developed CMV infection after HSCT (**Figure 9**).

**Figure 10:**
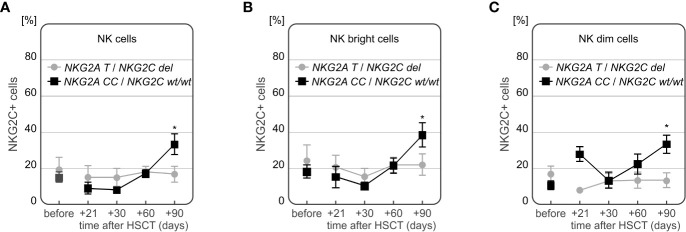
Differences in frequencies of NKG2C+ NK cells in patients with CMV infection carrying various *NKG2C* genotypes. Decreased frequency of NK cells expressing NKG2C receptor in recipients carrying *NKG2C* deletion (**A** *p=0.048, **B** *p=0.042 for NK cells and NK CD56bright subpopulation, respectively). Significantly higher frequency of NKG2C+ NK CD56dim cells in recipients with *NKG2A* rs7301582 *CC* and *NKG2C wt* genotype at +90 day after HSCT (p=0.005, **C**).

Interestingly, we also observed an association between the frequency of NKG2C+ NK cells and the presence of *NKG2C* deletion in the recipients. In patients who suffered from CMV infection and did not have *NKG2C* deletion, the frequency of NK cells expressing NKG2C increased to approach its maximum at day +90 after HSCT. Significant differences between recipients with and without *NKG2C del* variant were observed for the whole population of NK cells (p=0.048, **Figure 10A**), as well as for the two NK cell subsets – the NK CD56bright cells (p=0.042, **Figure 10B**), and NK CD56dim cells 90 days after HSCT (p=0.005, **Figure 10C**). Among the recipients with CMV infection after HSCT (n=12), half of them had the *NKG2A CC*/*NKG2C wt*/*wt* haplotype. Those recipients characterized with increased frequency of NKG2C+ NK cells, especially 3 months after HSCT (**Figure 10**). These associations were also seen when the patients positive for CMV IgG before transplantation were considered. There were 21 out of 28 (75%) seropositive recipients analysed.

## Discussion

Since GvHD and viral infections remain the most common HSCT complications, the need to develop new forms of treatment and characterize new prognostic as well as risk factors is a high priority. The HLA-E: NKG2A/C interaction may be a key element of these processes.

A number of studies report that donor and recipient HLA-E*01:03 homozygous genotype is related with better HSCT outcome due to a lower risk of acute and chronic GvHD development and a lower rate of relapse (48–53). However in contrast, in T-cell-replete transplantations, donor HLA-E*01:01/*01:03 genotype was associated with a higher transplant-related mortality and lower disease-free survival (36). Also, a study on kidney transplant patients showed that HLA-E*01:03 allele was correlated with a higher incidence of CMV infection after transplantation (37). A similar association was observed between HLA-E*01:03 and hepatitis C viral infection (38). This shows that results of previous studies on HLA-E polymorphism are somewhat inconsistent. Interestingly, our present study did not show any association between the presence of either HLA-E*01:03 or HLA-E*01:01 allele and a risk of post-transplant complications. However, we observed that HLA-E mismatch between donor and recipient is associated with a higher risk of CMV infection after HSCT. Likewise, an earlier study observed that HLA-E mismatch affected HSCT survival in patients with acute leukaemias, particularly in patients with advanced disease (54). In our previous studies, we also observed the unfavourable effect of HLA-E mismatching in patients after HSCT, such as increased acute GvHD risk (55–57). This suggests that HLA-E incompatibility, in addition to the presence or absence of specific alleles, may be an important factor affecting HSCT outcome.

Production of cell-free soluble HLA molecules is known to be implicated in cancer immune escape (58). HLA-E can likewise be present in a soluble form, resulting largely from proteolytic cleavage of membrane-bound HLA-E by matrix metalloproteinases (59). Increased soluble HLA-E concentration was associated with disease susceptibility in various disorders, including chronic hepatitis B, juvenile idiopathic arthritis, and gastric cancer (60–62). Soluble HLA-E is also involved in endothelial cell activation and may have a role in immunoregulatory functions of the endothelium (10). Furthermore, increased sHLA-E was detected in various different tumour cell culture supernatants and was proved to be upregulated by various cytokines, such as IFN-γ, IFN-α and TNF-α (11). Conversely, we showed that recipients with GvHD characterized with decreased serum sHLA-E levels 30 days after transplantation. This association was especially seen in patients with chronic GvHD, and to a lesser extent in patients with severe acute GvHD. Similarly, a recent study by Kordelas et al. showed that HSCT recipients who developed acute or chronic GvHD had lower sHLA-E levels up to one year after transplantation (35). These results suggest that decreased sHLA-E serum concentration may serve as prognostic factor for the development of GvHD with lower levels being associated rather with unfavourable prognosis. We also noticed that sHLA-E concentrations increased over time after HSCT, irrespective of the presence or absence of post-transplant complications. This observation might suggest that higher sHLA-E concentrations later on after transplantation may be related with better transplant outcome. Obviously this hypothesis requires further and more comprehensive studies.

Expression of the activating NKG2C NK cell receptor was shown to decrease a year after HSCT, following development of both acute and chronic GvHD in previous studies (39–41). This was not observed in our study, although it may be possible that this discrepancy was due to a shorter time of observation (3 months) of our patients. Conversely, NKG2C expression on NK cells is known to increase during CMV infection (19, 63), leading to emergence of potent mature NKG2A-/NKG2C+ CD56dim NK cells a year after transplantation (64). This is in accordance with our results showing a notable increase in NKG2C expression on all NK cell subtypes at the third month after transplantation as well as an increase of the unique NKG2A-/NKG2C+ NK cell population. The presence of the *NKG2C* deletion was previously reported to affect CMV reactivation after haploidentical HSCT or lung transplantation (28, 42, 43). We did not observe such an association. However, our study revealed that in patients with post-transplant CMV infection who carried at least one *NKG2C del* allele, the frequency of NKG2C+ NK cells was decreased in comparison to *wild type* homozygotes. This observation proves a functional association between the *NKG2C* gene polymorphism and its expression in patients with CMV infection. Interestingly, this finding seems to be confirmed in part by an earlier study on *NKG2C* deletion in CMV seropositive children (65). Although this association between *NKG2C* deletion and decreased NKG2C+ NK may be expected, there are studies indicating that it’s not always present (44).

Little is known about the *NKG2A* rs7301582 polymorphism. The *C* variant was reported to have a negative impact on response to anti-TNF treatment in Polish patients with rheumatoid arthritis (33). The present study is, to the best of our knowledge, the first to indicate the potential significance of *NKG2A* rs7301582 polymorphism in HSCT. It is uncertain how this SNP could exert its effect, since it is located in an NKG2A gene intron (intron 6). It is possible that this variant could affect splicing, although there is no evidence for it. Introns are known to affect gene expression by various indirect means, e.g. changing mRNA stability, influencing methylation/chromatin modifications, or harbouring cryptic splice sites (66, 67). Furthermore, SNPs can also act affect expression of other remote genes in a trans manner (68). Here, we confirm the role of *NKG2A* rs7301582 *C* allele as a negative factor since it occurred more frequently in recipients diagnosed with AML than in donor group.

Our study has, however, some limitations, i.e. small sample size, non-homogenous recipient group and lack of availability of all donor samples. Being aware of these limitations, we still are assured that our results make a strong contribution to the development of the field.

Taken together, the results obtained in this present study imply that the donor/recipient HLA-E mismatch is associated with a higher incidence of CMV infection after transplantation. Decreased serum sHLA-E concentration is associated with development of both chronic and acute GvHD in adult HSCT recipients. We also observed significant differences in the frequency of NKG2C expressing NK cells in the context of CMV infection. Recipients who were diagnosed with this complication were characterized by increased percentage of NKG2C+ NK cells. Moreover, we proved that *NKG2C* deletion was associated with expression level of the receptor, as patients carrying the *del* allele had a decreased frequency of NKG2C+ NK cells. Regarding the *NKG2A* polymorphism, the rs7301582 *C* allele may be associated with higher AML susceptibility. In conclusion, our results suggest that the sHLA-E level and expression of NKG2C on NK cells may act as a potential markers of post-transplant complications.

## Data availability statement

The datasets presented in this study can be found in online repositories. The data can be found at: https://cloud.hirszfeld.pl/index.php/s/sFaqtFN4wwiKWbK. Further inquiries can be directed to the corresponding author.

## Ethics statement

The studies involving humans were approved by Wroclaw Medical University Ethics Committee. The studies were conducted in accordance with the local legislation and institutional requirements. The participants provided their written informed consent to participate in this study.

## Author contributions

JS performed genotyping studies and assessment of sHLA-E concentration in serum samples, statistical analyses, drafted and finalized the manuscript; PŁ contributed to statistical analyses; DS performed flow cytometry experiments; AC, AS, MM, MS-K, WF, IS, BN-A, PS, MB, AT, GB, SG, TW provided patients’ clinical samples and clinical data; AC contributed to the conception of clinical data analysis; KB-K contributed to the conception and design of the study, data analysis, drafted and finalized the manuscript, and provided funding. All authors approved the final version of the manuscript.
