# Supplementary material for: Significance of HLA-E and its two NKG2 receptors in development of complications after allogeneic transplantation of hematopoietic stem cells
Source: Front Immunol. 2023 Oct 13;14:1227897. doi: 10.3389/fimmu.2023.1227897 (PMC10611459; doi:10.3389/fimmu.2023.1227897)
Supplement: Supplementary file 1 [file Table_1.docx]

**Supplementary**

**Table 1.** Distribution of genetic variants in AML and ALL patients and their donors.

|  |  | AML | | | ALL | | |
| --- | --- | --- | --- | --- | --- | --- | --- |
|  |  | **Recipients (n=85)** | **Donors (n=40)** | ***P* value** | **Recipients (n=27)** | **Donors (n=17)** | ***P* value** |
| *NKG2A* rs*7301582* | |  |  |  |  |  |  |
| *genotypes* | ***CC*** | 55 (64.71%) | 18 (45.00%) |  | 15 (55.56%) | 8 (47.06%) |  |
|  | ***CT*** | 27 (31.76%) | 19 (47.5%) |  | 10 (37.04%) | 5 (29.41%) |  |
|  | ***TT*** | 3 (3.53%) | 3 (7.50%) |  | 2 (7.41%) | 4 (23.53%) |  |
| *alleles* | ***C*** | 137 (80.59%) | 55 (68.75%) | 0.05321 | 40 (74.07%) | 21 (61.76%) | 0.24366 |
|  | ***T*** | 33 (19.41%) | 25 (31.25%) |  | 14 (25.93%) | 13 (38.24%) |  |
| *HLA-E rs1264457* | |  |  |  |  |  |  |
| *genotypes* | ***CC*** | 17 (20.00%) | 9 (22.50%) |  | 7 (25.93%) | 4 (23.53%) |  |
|  | ***CT*** | 35 (41.18%) | 19 (47.50% |  | 12 (44.44%) | 5 (29.41%) |  |
|  | ***TT*** | 33 (38.82%) | 12 (30%) |  | 8 (29.63%) | 8 (47.06%) |  |
| *alleles* | ***C*** | 69 (40.59%) | 37 (46.25%) | 0.41334 | 26 (48.15%) | 13 (38.24%) | 0.38718 |
|  | ***T*** | 101 (59.41%) | 43 (53.75%) |  | 28 (51.85%) | 21 (61.76%) |  |
| *NKG2C* deletion | |  |  |  |  |  |  |
| *genotypes* | ***wt/wt*** | 54 (63.53%) | 27 (67.5%) |  | 15 (55.56%) | 10 (58.82%) |  |
|  | ***wt/del*** | 30 (35.29%) | 12 (30%) |  | 10 (37.04%) | 5 (29.41%) |  |
|  | ***del/del*** | 1 (1.18%) | 1 (2.5%) |  | 2 (7.41%) | 2 (11.76%) |  |
| *alleles* | ***wt*** | 138 (81.18%) | 66 (82.5%) | 0.86252 | 40 (74.07%) | 25 (73.53%) | 1 |
|  | ***del*** | 32 (18.82%) | 14 (17.5%) |  | 14 (25.93%) | 9 (26.47%) |  |

**Table 2.** Distribution of genetic variants in HSCT recipients and healthy controls.

|  |  | Recipients | Controls^*^ | Controls^**^ |  |
| --- | --- | --- | --- | --- | --- |
| *NKG2A* rs*7301582* | |  | n=124 ^[1]^ | n=503 |  |
| *genotypes* | ***CC*** | 131(65.50%) | 84 (67.7%) | 330 (65.6%) |  |
|  | ***CT*** | 64 (32.00%) | 37 (29.8%) | 147 (29.2%) |  |
|  | ***TT*** | 5 (2.50%) | 3 (2,4%) | 26 (5.2%) |  |
| *alleles* | ***C*** | 326 (81.50%) | 205 (82.7%) | 807 (80.2%) |  |
|  | ***T*** | 74 (18.50%) | 43 (17.3%) | 199 (19.8%) |  |
| *HLA-E rs1264457* | |  | n=123 ^[2]^ | n=503 |  |
| *genotypes* | ***CC*** | 37 (18.5%) | 24 (20%) | 83 (16.5%) |  |
|  | ***CT*** | 92 (46%) | 60 (49%) | 269 (53.5%) |  |
|  | ***TT*** | 71 (35.5%) | 39 (32%) | 151 (30%) |  |
| *alleles* | ***C*** | 166 (41.5%) | 84 (46%) | 435 (43.2%) |  |
|  | ***T*** | 234 (58.5%) | 99 (54%) | 571 (56.8%) |  |
| *NKG2C deletion* | |  | n=259 ^[3]^ |  |  |
| *genotypes* | ***wt/wt*** | 134 (67%) | 185 (71%) |  |  |
|  | ***wt/del*** | 63 (31.5%) | 67 (26%) |  |  |
|  | ***del/del*** | 3 (1.5%) | 7 (3%) |  |  |
| *alleles* | ***wt*** | 331 (82.75%) | 437 (84%) |  |  |
|  | ***del*** | 69 (17.25%) | 81 (16%) |  |  |

^*^ European groups

^**^ The 1000 Genomes Project [1000 Genomes Project Consortium, Auton, A., Brooks, L. D., Durbin, R. M., Garrison, E. P., Kang, H. M., Korbel, J. O., Marchini, J. L., McCarthy, S., McVean, G. A., & Abecasis, G. R. (2015). A global reference for human genetic variation. Nature, 526(7571), 68–74. https://doi.org/10.1038/nature15393]

1. Iwaszko, M., Świerkot, J., Kolossa, K., Jeka, S., Wiland, P., & Bogunia-Kubik, K. (2016). Influence of CD94 and NKG2A variants on susceptibility to rheumatoid arthritis and efficacy of anti-TNF treatment. *Jt Bone Spine*, 83(1), 75–79. https://doi.org/10.1016/j.jbspin.2015.06.010
2. Sokolik, R., Gębura, K., Iwaszko, M., Świerkot, J., Korman, L., Wiland, P., & Bogunia-Kubik, K. (2014). Significance of association of HLA-C and HLA-E with psoriatic arthritis. *Hum Immunol*, 75(12), 1188–1191. https://doi.org/10.1016/j.humimm.2014.10.005
3. Toson, B., Michita, R. T., Matte, M. C. T., Soares, R., Lawisch, G. K. S., Mattevi, V. S., & Chies, J. A. B. (2022). Assessment of NKG2C copy number variation in HIV-1 infection susceptibility, and considerations about the potential role of lacking receptors and virus infection. *J. Hum. Genet.*, 67(8), 475–479. https://doi.org/10.1038/s10038-022-01029-w

**Table 3.** Results of a multivariate logistic regression analysis of CMV infection risk.

|  | odds ratio (95% CI) | p-value |
| --- | --- | --- |
| HLA-E mismatch | 5.92 (1.57 – 29.22) | **0.014** |
| NKG2C genotype | 0.82 (0.28 – 2.30) | 0.703 |
| recipient’s age | 0.98 (0.94 – 1.01) | 0.192 |
| recipient CMV status | 35.90 (4.89 – 812.49) | **0.003** |
| donor CMV status | 0.65 (0.19 – 2.19) | 0.489 |
| recipient’s sex | 0.57 (0.21 – 1.55) | 0.276 |
| donor’s sex | 1.22 (0.46 – 3.35) | 0.693 |
| HLA mismatch | 1.36 (0.49 – 3.89) | 0.557 |
